# Role of Alpha-actinin-3 in Contractile Properties of Human Single Muscle Fibers: A Case Series Study in Paraplegics

**DOI:** 10.1371/journal.pone.0049281

**Published:** 2012-11-08

**Authors:** Siacia Broos, Laurent Malisoux, Daniel Theisen, Marc Francaux, Louise Deldicque, Martine A. Thomis

**Affiliations:** 1 Exercise Physiology Research Group, Department of Kinesiology, Faculty of Kinesiology and Rehabilitation Sciences, KU Leuven, Heverlee, Belgium; 2 Physical Activity, Sports and Health Research Group, Department of Kinesiology, Faculty of Kinesiology and Rehabilitation Sciences, KU Leuven, Heverlee, Belgium; 3 Faculté d’Éducation Physique et de Réadaption, Faculté de Médecine, Université Catholique de Louvain, Louvain-la-Neuve, Belgium; 4 Sports Medicine Research Laboratory, Public Research Center for Health, Grand-Duchy of Luxembourg, Luxembourg; Sanjay Gandhi Medical Institute, India

## Abstract

A common nonsense polymorphism in the *ACTN3* gene results in the absence of α-actinin-3 in XX individuals. The wild type allele has been associated with power athlete status and an increased force output in numeral studies, though the mechanisms by which these effects occur are unclear. Recent findings in the *Actn3^−/−^* (KO) mouse suggest a shift towards ‘slow’ metabolic and contractile characteristics of fast muscle fibers lacking α-actinin-3. Skinned single fibers from the quadriceps muscle of three men with spinal cord injury (SCI) were tested regarding peak force, unloaded shortening velocity, force-velocity relationship, passive tension and calcium sensitivity. The SCI condition induces an ‘equal environment condition’ what makes these subjects ideal to study the role of α-actinin-3 on fiber type expression and single muscle fiber contractile properties. Genotyping for *ACTN3* revealed that the three subjects were XX, RX and RR carriers, respectively. The XX carrier’s biopsy was the only one that presented type I fibers with a complete lack of type II_x_ fibers. Properties of hybrid type II_a_/II_x_ fibers were compared between the three subjects. Absence of α-actinin-3 resulted in less stiff type II_a_/II_x_ fibers. The heterozygote (RX) exhibited the highest fiber diameter (0.121±0.005 mm) and CSA (0.012±0.001 mm^2^) and, as a consequence, the highest peak force (2.11±0.14 mN). Normalized peak force was similar in all three subjects (*P* = 0.75). Unloaded shortening velocity was highest in R-allele carriers (*P*<0.001). No difference was found in calcium sensitivity. The preservation of type I fibers and the absence of type II_x_ fibers in the XX individual indicate a restricted transformation of the muscle fiber composition to type II fibers in response to long-term muscle disuse. Lack of α-actinin-3 may decrease unloaded shortening velocity and increase fiber elasticity.

## Introduction

Human physical performance is considered a complex phenotype determined by genetic potential and variation in athletic ability has long been recognized as having a strong heritable component [1]. One of the more commonly investigated genes in this area is *ACTN3*. This gene encodes for α-actinin-3 that is mainly expressed in type II muscle fibers. These fibers are responsible for the generation of rapid forceful contractions, but are less resistant to fatigue and eccentric damage [2]. As a sarcomeric protein, α-actinin-3 is a key factor for stabilization and integrity of the muscle contractile apparatus by cross-linking actin and other structural components at the Z-disk [3].

A common single nucleotide polymorphism (SNP) at codon 577 (rs1815739) of the *ACTN3* gene results in a premature stopcodon. Homozygotes for the null polymorphism are completely deficient for α-actinin-3 and represent ∼16% of the Caucasian population (RX = 48%; RR = 36%) [2]. Interestingly, this deficiency does not result in a disease phenotype or disruption of sarcomere formation and structure. However, the *ACTN3* R577X polymorphism has been associated with athletic status and muscle phenotypes in several ethnicities. A number of independent studies have found an overrepresentation of the R-allele in power athletes compared to the general population [4]–[7]. In non-athletic individuals, the presence of the R-allele increases the capacity to perform high power muscle contractions, whereas α-actinin-3 deficiency is detrimental to muscle strength and sprinting performance [8]–[10]. A meta-analysis performed on the current data found an association between the R-allele and sprint/power athletic status in Europeans [11].

The influence of the SNP on muscle power suggests a role for α-actinin-3 in skeletal muscle optimization, including the regulation of muscle metabolism and skeletal muscle fiber differentiation. The generation of the *Actn3^−/−^* knockout (KO) mouse provided a first insight into the effects of α-actinin-3 deficiency on skeletal muscle function [12]. Compared to wild type (WT) mice, the KO mice show lower muscle mass, muscle strength, II_b_ fiber diameter and an enhanced recovery from fatigue. In addition, KO fast muscle fibers have an increased activity of aerobic enzymes and a delayed Ca^2+^ loading of the sarcoplasmic reticulum [13]–[15]. The latter implies the development of more slow-twitch and oxidative characteristics in fast fibers in the absence of α-actinin-3. Our research group already explored the change towards more pronounced oxidative characteristics in α-actinin-3 deficient fast fibers by testing a set of oxidative markers in human muscle. No difference was found in cytochrome c oxidase and succinate dehydrogenase staining in fast fibers between XX and RR genotype groups [16].

Alterations in muscle fiber contractile characteristics due to the R577X polymorphism have not yet been explored in humans. As α-actinin-3 is almost exclusively expressed in type II fibers, testing subjects with high percentages of fast fibers would be most favorable. Individuals with spinal cord injury (SCI) are characterized by a chronic disuse of the affected limb muscles and have a predominance of type II fibers in the paralyzed muscles. The SCI condition also induces an ‘equal environment condition’ between subjects that is hard to establish in able-body subjects. The study of muscle tissue in SCI subjects with different *ACTN3* R577X genotype seems therefore ideal to study the role of α-actinin-3 on fiber type expression and single muscle fiber contractile properties.

Long-term disuse of the limbs induces several structural changes in the immobilized muscle [17]. Most prominent is a rapid and significant loss of muscle mass as a result of a reduction in fiber diameter, coupled with a loss of muscle fibers, especially type I fibers. The remaining slow fibers undergo a transformation to the fast fatigable fibers which begins between four and seven months post-SCI. In the paralyzed quadriceps muscle, the fiber-type transformation reaches a steady state after about 70 months and the muscle is composed predominantly of fast MHC isoforms [18]. As a consequence of the changed muscle fiber composition, SCI patients demonstrate a change in muscle contractile properties [17]. In both complete and incomplete chronic SCI patients a decrease in force is apparent in all paralyzed muscles [19], [20]. The speed-related changes show shorter half-relaxation time and faster time to peak contraction [21]. Malisoux et al. (2007) found the unloaded shortening velocity and normalized peak power to be higher in fast fibers from paralyzed muscles compared to able-bodied controls.

To explore the differences in contractile properties in muscle fibers with different *ACTN3* genotype, single fiber contractile characteristics were determined in three men with SCI, one with the RR, RX and XX genotype respectively. Velocity and force of all single muscle fiber types were measured. Consistent with a role for α-actinin-3 in the generation of rapid forceful contractions, a higher force and velocity is expected in fast fibers of R-allele carriers. In type I fibers, we predict similar results in all three subjects as α-actinin-3 is only expressed in type II fibers. In accordance with the lower percentage of type II_x_ fibers in XX homozygotes, we hypothesize a reduced transformation towards completely fast-twitch muscles in the SCI individual with α-actinin-3 deficiency [8]. Another factor that contributes to the differentiation between fast and slow fibers is the calcium sensitivity of the contractile proteins. In line with the observation of slow twitch characteristics in *Actn3^−/−^* fast fibers, we hypothesize an increased sensitivity for calcium in fast fibers of the XX SCI individual. Hysteresis and Young’s Modulus were measured to determine muscle fiber elasticity. α-actinin-3 deficiency has been linked with increased susceptibility to eccentric contraction-induced damage in human [22] and mice muscle [23]. Differences in Z-disk rigidity and overall fiber elasticity might contribute to this difference in susceptibility and therefore genotype-dependent differences in hysteresis and Young’s Modulus were hypothesized.

## Participants and Methods

### 1. Participants

Single fiber data was obtained from three men with chronic SCI (age 38±5 yr, height 182±2 cm, body mass 95±2 kg) who volunteered to participate in a study of Malisoux et al. (2007). Baseline characteristics of the three test subjects are available in Table 1. The SCI subjects were not able to stand or walk and had all been permanent wheelchair users for a minimum of 17 yr (23±3 yr). *SCI 1* and *2* had a complete motor paralysis as a consequence of C7 and D7/8 lesions, respectively. *SCI 3* had motor incomplete lesions (level L1) and was able to have voluntary although not functionally useful contractions of his quadriceps muscles. All participants were informed of the risks associated with the investigation and provided written, informed consent. The protocol of the original study had been previously approved by the Faculty Ethical Review Committee and complied with the principles of the Declaration of Helsinki. A separate informed consent to collect saliva for DNA analysis was signed by all included participants.

**Table 1:** Baseline characteristics of test subjects.

		Age (yr)	Weight (kg)	Height (cm)	TSI (yr)
SCI 1	RR	48	93	179	17
SCI 2	RX	43	93	186	24
SCI 3	XX	28	100	181	28

TSI = Time since injury.

### 2. Muscle Biopsies

The needle biopsy method with suction was used to obtain a sample from the vastus lateralis of the right leg. Muscle samples were immediately placed in cold (0°C) skinning solutions [24] and sectioned longitudinally in small bundles of fibers. The bundles were stored in regularly replaced skinning solution at –20°C for at least 5 days before the first experiment. Composition of all solutions can be found in Malisoux et al. (2007).

### 3. Single Muscle Fiber Set-up

After skinning, single muscle fibers were evaluated for either P_0_, V_0_, and force-velocity relationship or passive tension characteristics on a dynamic force set-up. An isometric set-up was used to determine calcium sensitivity. Detailed protocols have been previously described [24], [25].

### 4. Single Muscle Fiber Analysis

All single muscle fiber analyses were performed as previously described by Malisoux et al. 2007. In short, single fiber dimensions including fiber length and cross-sectional (CSA) were determined on a numeric picture of the fiber after adjustment of sarcomere length to 2.5 µm. Peak activated force (P_0_, mN) was determined as the stable maximal force developed by the fiber while submerged in activating solution (pCa 4.5). The unloaded shortening velocity (V_0_) was measured by the slack test; each fiber was maximally activated and then rapidly shortened, such that force fell to baseline and redeveloped after a time lapse, proportional to the step length. Fiber *V_0_* was determined as the slope of the fitted line and was expressed in fiber lengths (FL) per second to account for differences in the number of sarcomeres in series between different fibers tested. To determine force-velocity relationship, the fiber was subjected 5–6 times to three successive isotonic load clamps. The data obtained on a single fiber were fitted using an iterative nonlinear curve-fitting procedure (Marquardt-Levenberg algorithm) based on the Hill equation. Passive tension was tested with a progressive stretch-release protocol while the fiber remained in the pCa 9.0 solution. Young's modulus (kN/m^2^) and hysteresis (kN/m^2^) were calculated to assess visco-elastic properties of the fiber. To construct the force-calcium relationship, the fiber was transferred rapidly from the relaxing solution to different activating solutions (pCa 4.5 to 6.4). Experimental data points were fitted with the Hill equation P_r_ = pCa^n^/(pCa_50%_ *^n^* + pCa*^n^*), where pCa_50%_ is the Ca^2+^ concentration at which half-maximal activation occurs and *n* is the Hill coefficient, an indicator of the slope of the relationship.

### 5. Fiber MHC Isoform Determination

**Figure 1:**
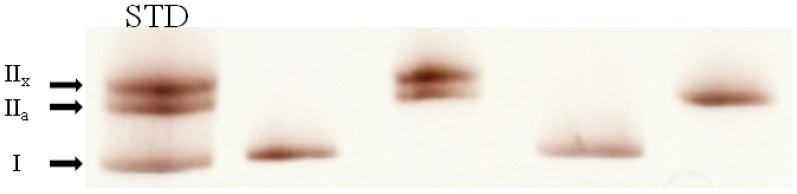
MHC isoform determination in single muscle fibers using silver staining. The figure is a representative example of an 8% silver stained SDS gel. Samples from single fibers containing (from left to right) the type I, II_a_/II_x_, I and II_a_ MHC isoform, respectively. STD, standard samples containing MHC I, II_a_, and II_x_ isoforms.

After completion of the mechanical tests, the fiber segment was dissolved in 25 µl of SDS sample buffer, and stored at –20°C until analyzed for MHC isoform content using SDS-PAGE as previously described [26]. In short, the sample was heated at 95°C for 3 min, and 5 µl of this extract was loaded on a electrophoresis system (Bio-Rad, Hercules, CA) with a 4% (wt/vol) acrylamide stacking gel and 8% separating gel. Following gel electrophoresis at 140 V for 12 h at 4°C, gels were silver stained as described by Giulian et al. [27] to identify MHC isoform based on migration position (Fig. 1). The MHC expression was determined on each fiber segment used for the mechanical tests plus on ∼50 fibers per biopsy to determine the MHC profile on some 100 fibers for each subject.

### 6. Genotyping

DNA was extracted from saliva collected in an Oragene DNA self-collection kit (Oragene, Ontario, Canada) according to the protocol provided by the manufacturer. The *ACTN3* R577X polymorphism genotyping was performed using a TaqMan SNP genotyping assay (ID C__590093_1, Applied Biosystems). All reactions were set up manually, and allele calling was done using SDS 1.3 software and visually checked.

### 7. Statistical Analysis

Explorative analyses were performed by ANOVA to compare single fiber characteristics between ACTN3 R577X genotypes (Tukey HSD post-hoc tests). As hybrid type II_a_/II_x_ fibers were the only fiber type present in all three individuals, differences between the paraplegics in single fiber dimensions and contractile properties could only be analyzed in these fibers. *P*<0.05 were interpreted as significant differences.

## Results

### Muscle Fiber Composition

**Figure 2:**
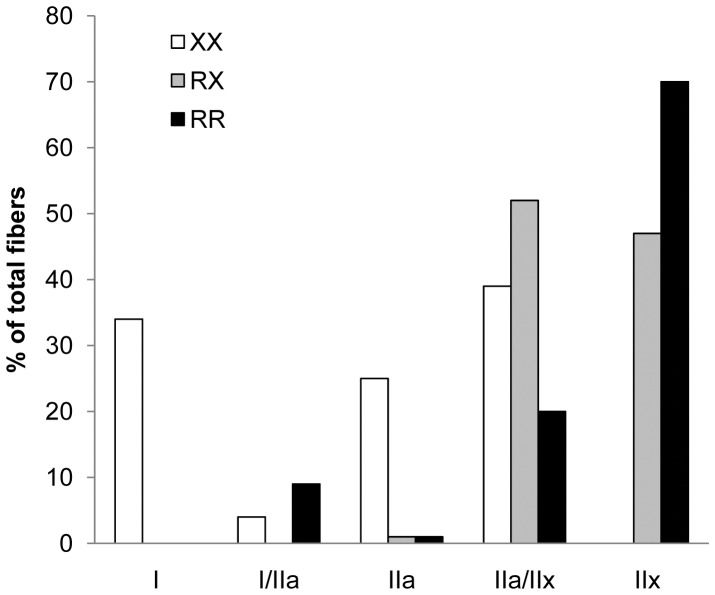
MHC isoform profiles of single fibers from the three SCI subjects.

MHC isoform profiles were established based on a total of 317 single fibers. As illustrated in Fig. 2, type I fibers were only present in the XX individual (XX = 34%; RX, RR = 0%). Additionally, type II_a_ fibers were less expressed in the two R-allele carriers (XX = 22%; RX, RR = 1%). The proportion of type II_x_ fibers increased with each additional R-allele (XX = 0%; RX = 47%; RR = 70%).

### Single Fiber Dimensions

Fiber diameter and CSA were determined on 5 (RR), 15 (RX) and 11 (XX) type II_a_/II_x_ fibers. Both were found to be the highest in the heterozygote (Table 2).

**Table 2:** Single muscle fiber hybrid type IIa/IIx dimensions and contractile properties over ACTN3 genotypes in paraplegics.

	RR	RX	XX	*P*-value
Dimensions				
Diameter, mm	0.073±0.010	0.121±0.005	0.090±0.006	<0.001^a^
CSA, mm^2^	0.004±0.002	0.012±0.001	0.006±0.001	<0.001^a^
Dynamic				
P_0_, mN	0.96±0.25	2.11±0.14	1.29±0.17	<0.001^a^
P_0_/CSA, kN/m^2^	159±13	169±8	160±9	0.75
V_0_, FL/s	6.82±0.48	6.61±0.28	5.19±0.31	0.002^b^
Young’s Modulus, kN/m^2^	41.4±2.4	23.5±1.3	14.2±1.7	<0.001^c^
Hysteresis, kN/m^2^	2.45±0.24	1.24±0.14	0.56±0.17	<0.001^c^
Isometric				
pCa_50%_	5.63±0.03	5.72±0.03	5.73±0.03	0.10
Act. Tresh.	6.82±0.06	6.78±0.05	6.76±0.05	0.74
*n1*	1.91±0.17	1.90±0.12	1.85±0.12	0.94
*n2*	2.12±0.15	2.43±0.11	2.52±0.11	0.12

Values are means ± SE.

a RX > RR and XX;

b RR and RX > XX;

c RR > RX > XX.

### P_0_, V_0_ and Force-velocity Relationship

These analyses were assessed on a total of 31 type II_a_/II_x_ fibers, respectively 5 (RR), 15 (RX) and 11 (XX) fibers. Maximal force values were highest in the RX individual (*P*<0.001). As force highly depends on the number of sarcomeres in parallel, normalized force was calculated by dividing P_0_ by the CSA of the respective fiber. No statistically significant difference was observed between the three individuals for normalized single fiber force (*P* = 0.75), nor in force-velocity curve pattern. As hypothesized, maximal unloaded velocity (V_0_) was significantly higher in the two R-allele carriers (Table 2).

### Passive Tension

**Figure 3:**
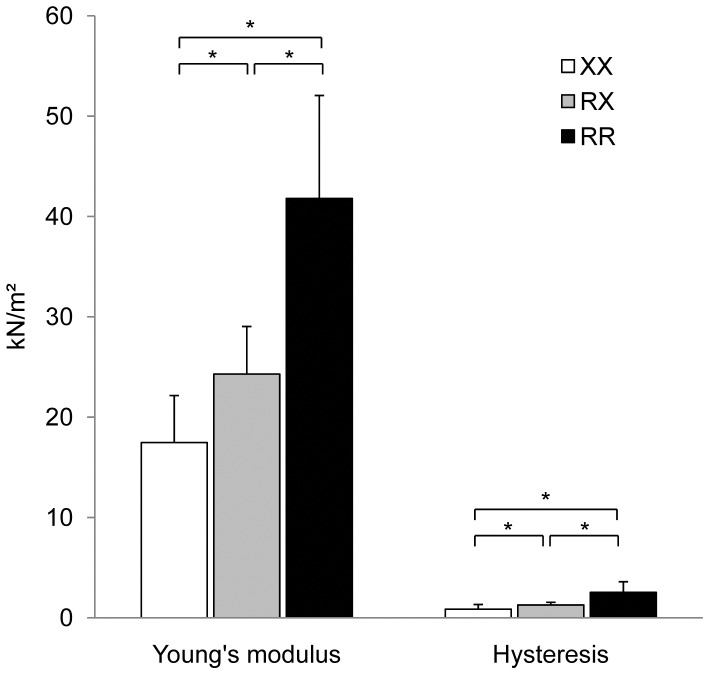
Average Young’s modulus and hysteresis of type II_a_/II_x_ fibers over genotypes. Error bars are SD. *Statistical difference at *P*<0.001.

Passive characteristics of single fibers were evaluated on 3 (RR), 10 (RX) and 6 (XX) type II_a_/II_x_ fibers. A significant increase was found for values of complex Young’s modulus for each additional R-allele present (*P*<0.001). Hysteresis increased in a similar pattern (*P*<0.001) (Fig. 3; Table 2).

### Force-pCa Relationship

**Figure 4:**
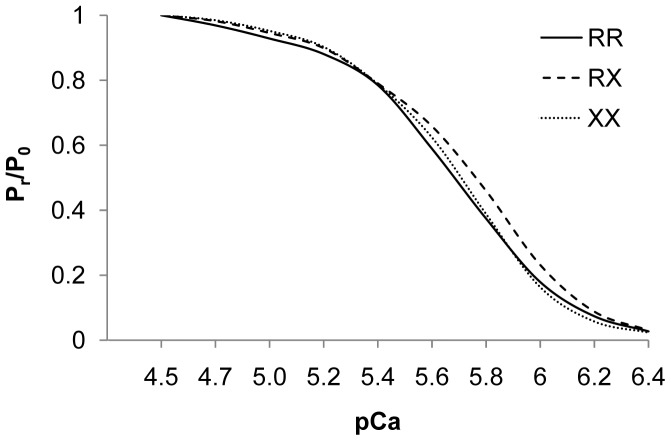
Force-pCa curve of the type II_a_/II_x_ fibers of each subject. P_r_/P_0_, force expressed relative to maximal Ca^2+^-activated force, plotted against the respective Ca^2+^ concentrations expressed in pCa (−log [Ca^2+^]). pCa = 4.5 contains the highest Ca^2+^ concentration.

Force-pCa relationship was determined based on 23, 23 and 14 type II_a_/II_x_ fibers of *SCI 1*, *2* and *3* respectively. The force-pCa curve did not differ between fibers of the different genotypes. Also, the Ca^2+^ activation threshold was similar for all three individuals. No differences appeared for pCa_50%_ and Hill plot coefficients *n*_1_ and *n*_2_ (Fig. 4; Table 2).

## Discussion

Three individuals with spinal cord injury were included in this study to explore the influence of alpha-actinin-3 deficiency on contractile properties of individual skeletal muscle fibers. Long term paralysis induced a ‘standardized’ environmental/disused status of the vastus lateralis muscle in all three subjects.

The most striking change in the chronic immobilized muscle is the transformation of the muscle composition towards fast fibers (MHC isoform II_a_ and II_x_) to the detriment of type I fibers. Our results indicate a limitation in a shift in muscle composition towards type II_x_ fibers and in the disappearance of type I fibers as a result of long term muscle unloading in the absence of α-actinin-3. The XX homozygote was the only participant with preservation of type I fibers (35%), while no II_x_ fibers were found in this individual. In contrast, the paralyzed muscle of the RR and RX individual was composed out of respectively 71% and 43% type II_x_ fibers. These data suggest a role for α-actinin-3 in the regulation of fiber type expression. This is in accordance with Vincent et al. (2007) who found an increased percentage of fast glycolytic type II_x_ fibers in RR homozygotes compared to XX individuals [8]. The promotion of the formation of fast twitch fibers by α-actinin-3 could be regulated through a binding of the protein with calsarcins that interact with the signaling protein calcineurin. Thus, the effects of the SNP on athletic/muscle performance might rely, at least in part, on the regulation of fiber type proportions by α-actinin-3. However, this would not exclude a role for other interactions or signaling pathways in the phenotypic consequences of α-actinin-3 deficiency.

Differences in fiber stiffness were found between the three test subjects. The visco-elasticity of the fiber as measured by hysteresis increased with each additional R-allele. A similar trend was found for complex Young’s modulus. The latter represents the intrinsic mechanical properties of the muscle fiber. Therefore, a change in Young’s modulus corresponds to a fundamental reorganization of load-bearing proteins in the skeletal muscle. The most obvious candidate protein is the giant intramuscular molecule titin that connects the myosin filament to the Z-disk [28]. This protein is elastic and bears passive tension in the skeletal muscle fiber, thus contributing substantially to the total passive stiffness of the muscle [29]. Variations in titin isoforms are major determinants of passive stiffness of the sarcomere, especially in fast muscles [30], [31]. More specifically, variability in the size of titin has been shown to relate to alterations in skeletal muscle stiffness [32]. Since titin is the major source of intracellular passive tension, a modification in titin isoforms, concentration or organization in the muscle fiber can explain a change in fiber elasticity. Recently, Seto et al. (2011) showed a preference for titin to interact with α-actinin-2 instead of α-actinin-3 [23]. In the absence of α-actinin-3, the higher binding affinity of titin to α-actinin-2 likely changes the concentration and organization of titin in the muscle fiber which results in an increased fiber elasticity. In skinned rat fibers, a decrease in Young’s modulus has been associated with a loss of the relative amount of titin [33]. These data suggests the increased elasticity in α-actinin-3 deficient fibers, as determined by a decrease in Young’s modulus and hysteresis, to be due to a change in titin concentration or organization as a result of the preference of titin to interact with α-actinin-2.

By increasing its stiffness, the muscle fiber may develop a greater ability to resist mechanical stress [34]. Thus, the higher binding affinity of titin to α-actinin-2 in XX fibers could decrease the capacity of the Z-disk to withstand eccentric stress in these fibers. Evidence for a possible role for α-actinin-3 in protecting the fast fiber from eccentric damage has been found in humans and mice. After a single eccentric exercise bout, XX individuals had higher pain scores than RR individuals. The serum creatine kinase activity, an indirect indicator for muscle damage, tended to be higher in α-actinin-3 deficient muscles. In addition, relative torques post-eccentric contraction was lower in the XX group [22]. Force deficit is an indirect measure of the magnitude of contraction-induced damage. Thus, the higher force deficits after an eccentric bout in XX homozygotes may reflect greater susceptibility to damage in α-actinin-3 deficient fibers. The basis for increased force deficit and susceptibility to damage is generally attributed to changes in proteins located at the Z-disk [35]. In line, the *Actn3^−/−^* KO mouse shows higher expression of Z-disk associated proteins γ-filamin, myotilin, desmin, αB-crystallin and ZASP, as well as genes associated with muscle growth and regeneration after eccentric damage [23], which suggests an increased need for muscle remodeling and regeneration as a result of ongoing damage in the α-actinin-3 deficient fibers. As type II fibers are more susceptible to eccentric stress compared to type I fibers, the protection to damage by α-actinin-3 is an important mechanism during eccentric contractions. This could, in part, explain the lack of athletes with the XX genotype in sports such as sprinting.

It was expected that the RR individual would have the thickest fibers as the reduced muscle mass observed in XX homozygotes [36] is primarily caused by a decrease in cross-sectional area (CSA) of fast fibers. The *Actn3^−/−^* KO mouse model shows no change in the total number of muscle fibers whilst the CSA of type II_b_ fibers in KO mice were found to be 34% smaller compared to II_b_ fibers in WT mice [37]. Nevertheless, looking at dimensions of type II_a_/II_x_ fibers in these three paraplegics, the diameter and the CSA were smallest in the RR individual with highest values in the heterozygote. As force highly depends on the number of sarcomeres in parallel, it was not surprising that the raw force values were highest in the RX individual. After correction for fiber CSA, no more differences were found in peak force between the three subjects.

A switch to more slow twitch and oxidative characteristics is observed in fast KO fibers [13], [37], which suggests an increased calcium sensitivity in these fibers. The pCa activation threshold, pCa_50%_ and Hill plot coefficient *n_1_* and *n_2_* can be used as an indication for the sensitivity of the contractile proteins to calcium. In this study, none of these three parameters differed over genotypes. Consequentially, calcium sensitivity can be assumed to be similar in all fast fibers. Likewise, force-pCa curves followed the same pattern in all three individuals. This is in accordance with Chan et al. (2011) who found no difference in calcium sensitivity between WT and KO fast muscle fibers.

A limitation of this study is the inclusion of a SCI patient with motor incomplete lesions who was able to have voluntary although not functionally useful contractions of his quadriceps muscles. No studies are available on differences in muscle fiber composition between complete and incomplete SCI patients. Yet there are studies to show similar percentages of muscle fiber types in the quadriceps muscle of complete SCI patients as noted in the XX homozygote [38]. The reason for the preservation of type I fibers in these patients was uncertain. The *ACTN3* R577X polymorphism may, partially, explain the differences in muscle fiber composition seen between SCI patients. Still, the total number of fibers tested per individual is relatively low. Therefore these results should be considered as preliminary, particularly since this study comprises only three subjects. Furthermore, the three subjects are now contrasted according to a specific functional gene polymorphism, resulting in the presence or absence of the alpha-actinin-3 protein in the Z-lines of fast muscle fibers. However, other gene variants between the three subjects might contribute to the observed differences in the fiber characteristics through other pathways or modified myofibrillar proteins. Also, previous findings state that muscle composition and contractile properties remain constant after about 70 months of immobilization. Therefore, the substantial difference in TSI between the three test subjects is not considered to be a confounding factor.

In conclusion, this exploratory study demonstrated for the first time the effects of the *ACTN3* R577X polymorphism on contractile properties of single muscle fibers in humans, with a specific application in paraplegic subjects. Absence of α-actinin-3 resulted in less stiff hybrid type II_a_/II_x_ fibers, which may increase susceptibility to eccentric stress damage. No difference was found in calcium sensitivity or force-pCa curve across genotypes. Contrary to our expectation, the heterozygote (RX) exhibited the highest fiber diameter and CSA. The unloaded shortening velocity was highest in R-allele carriers. The preservation of type I fibers and the absence of type II_x_ fibers in the XX individual indicate a restricted transformation of the muscle fiber composition to type II fibers in response to long-term muscle disuse. Our results provide insights into the underlying mechanisms by which the *ACTN3* R577X induces its effects on muscle phenotypes.
